# Synthetic Cannabinoids Influence the Invasion of Glioblastoma Cell Lines in a Cell- and Receptor-Dependent Manner

**DOI:** 10.3390/cancers11020161

**Published:** 2019-01-31

**Authors:** Tim Hohmann, Kerstin Feese, Thomas Greither, Chalid Ghadban, Vivian Jäger, Faramarz Dehghani, Urszula Grabiec

**Affiliations:** 1 Institute of Anatomy and Cell Biology, Medical Faculty of Martin Luther University Halle-Wittenberg, Grosse Steinstrasse 52, 06108 Halle (Saale), Germany; tim.hohmann@medizin.uni-halle.de (T.H.); kerstin.feese@posteo.de (K.F.); chalid.ghadban@medizin.uni-halle.de (C.G.); jaeger.vivian@gmx.de (V.J.); urszula.grabiec@medizin.uni-halle.de (U.G.); 2 Center for Reproductive Medicine and Andrology, Martin Luther University Halle-Wittenberg, 06108 Halle (Saale), Germany; thomas.greither@uk-halle.de

**Keywords:** glioblastoma, cannabinoids, micro-RNA, invasiveness, pAkt

## Abstract

The current treatment of glioblastoma is not sufficient, since they are heterogeneous and often resistant to chemotherapy. Earlier studies demonstrated effects of specific cannabinoid receptor (CB) agonists on the invasiveness of glioblastoma cell lines, but the exact mechanism remained unclear. Three human glioblastoma cell lines were treated with synthetic CB ligands. The effect of cannabinoids on microRNAs (miRs), Akt, and on the expression of proliferation and apoptosis markers were analyzed. Furthermore, in a model of organotypic hippocampal slice cultures cannabinoid mediated changes in the invasiveness were assessed. MicroRNAs and the activation of Akt which are related to cell migration, apoptosis, and proliferation were evaluated and found not to be associated with changes in the invasiveness after treatment with CB ligands. Also proliferation and/or apoptosis were not altered after treatment. The effects of cannabinoids on invasiveness could be blocked by the application of receptor antagonists and are likely mediated via CB_1_/CB_2_. In conclusion, our results suggest that cannabinoids can influence glioblastoma cell invasion in a receptor and cell type specific manner that is independent of proliferation and apoptosis. Thus, cannabinoids can potentially be used in the future as an addition to current therapy.

## 1. Introduction

Glioblastoma is one of the most devastating tumors with a median survival time of 18 months [1]. The standard therapy includes surgical dissection and an adjuvant radio- and chemotherapy. However, the current treatment is not sufficient, since glioblastomas are very heterogeneous and often radio- and chemoresistant [1]. This heterogeneity is reflected by different cells-of-origin and molecular subtypes [2]. Furthermore, the number of mutations carried by glioblastomas can include up to approximately 1700 mutated genes, leading to the introduction of different glioblastoma subclasses based on their histology and molecular features [2]. Subsequently, the cells in individual glioma differ in their behavior regarding invasiveness, motility, morphology, genetics, susceptibility to therapy, and their ability to form tumors [3]. The resulting heterogeneous population of glioblastomas gives rise to the need for an improved understanding of tumor spreading and better or individualized therapeutic options. One potential novel therapeutic target might be the endocannabinoid system. Endocannabinoids are lipids, synthetized on demand, activating the G protein coupled cannabinoid receptors (CB)_1_ and CB_2_ [4]. Decades ago cannabinoids were shown to exert antineoplastic activities [5]. Current literature implies a potential for cannabinoids as anticancer agents [6,7,8]. Cannabidiol (CBD) and Δ-9-tetrahydrocannabinol (THC) are typical phytocannabinoids and were used in the majority of studies; however, the involvement of cannabinoid receptors in pharmacological actions beyond those phytocannabinoids remains unclear [9,10]. Different phyto- and synthetic cannabinoids were shown to reduce proliferation, angiogenesis, and to induce apoptosis in various tumor types including glioblastoma cells [7]. Δ-9-tetrahydrocannabinol and CBD were reported to exert a multitude of antitumorous effects on U-87 MG cells [11,12,13]. Furthermore, THC was shown to induce apoptosis of primary brain tumor cells [11]. In U-87 MG cells, THC did not reduce cell viability but tumor volume [12]. A combined administration of THC and CBD decreased growth of U-87 MG cell derived tumor xenografts via apoptosis [12] and inhibited cell cycle in SF126 and U-251 MG cells, but not in U-87 MG, and induced apoptosis in U-251 MG cells [14]. Synthetic cannabinoids like the agonists ACEA, JWH133 or antagonists/inverse agonists AM281 and AM630 are known to be more selective than phytocannabinoids [4]. A previous study in our lab demonstrated that the invasiveness of U-87 MG was not affected by treatment with specific CB_1_ and CB_2_ agonists, while the CB_2_ agonist JWH133 increased the invasiveness in U-138 MG and had an inverse effect on LN229 cells. The CB_1_ agonist ACEA had an anti-invasive effect in U-138 MG cells but none in LN229 [15]. Glioblastoma cells seem therefore to respond in different ways to cannabinoid treatment. One potential pathway that might be involved in the antitumoral effects of cannabinoids is the Akt pathway [10].

An association between cannabinoids and Akt signaling was shown before in astrocytoma [13,14,16]. Phosphatidylinositol-3-kinase (PI3K)-Akt signaling modulates cell survival, prevents apoptosis by phosphorylation and inactivates the proapoptotic Bcl-2 family protein, BAD [17]. Interestingly, in a murine astrocytoma line (murine delayed brain tumor cell line) only clones expressing low levels of CB_1_ and CB_2_ underwent ERK 1/2 induced apoptosis after cannabinoid treatment. Conversely, no cell death was observed in tumor cells with high CB_1_ and CB_2_ expression. As a main reason the coupling of cannabinoid receptors to the prosurvival signaling pathway Akt was assumed [16]. Additionally, CBD and THC were shown to inhibit the phosphorylation of Akt in U-87 MG cells after 24 h [13,18]. 

While the role of pAkt, various cannabinoids, and microRNAs (miRs) in glioblastoma seems to be established, hardly anything is known if and how cannabinoids affect miRs in these tumors. Multiple miRs are associated with pro- or antitumorous actions. One of the best examined miR is miR-21 targeting several proapoptotic key factors, affecting invasion, chemoresistance, proliferation, and tumor growth [19,20,21,22,23,24]. Expression of miR-27a was shown to correlate with grading in astrocytoma, to have a prognostic value, and to suppress migration of glioblastomas [25,26]. miR-34a and miR-210 were shown to be regulators of cell proliferation [27] and miR-423-5p contributed to a malignant phenotype and temozolomide chemoresistance in glioblastomas, promoted tumorigenicity, angiogenesis, invasion, and conferred the resistance to therapy [28]. Additionally, some miRs (e.g., miR-21) exert antitumoral effects in glioblastomas via the suppression of Akt activity [29]. In some systems interactions between cannabinoid receptors and miRs were observed [30,31,32,33,34,35], but while there is a large amount of data on the effect of miRs on glioblastomas, studies about the interaction of cannabinoids with miRs are scarce.

Taken together studies exploring the putative anti-invasive properties of synthetic cannabinoids in glioma cells are still limited and the molecular mechanisms underlying their effect are poorly understood. The aim of this study was to explore possible molecular and cellular explanations for biological effects of cannabinoids on the invasive capacity of glioblastoma cells as observed in a previous study [15]. Therefore, the expression of different miRs and the activation of Akt which are related to tumor progression and proliferation after cannabinoid application were examined. Furthermore, we aimed at characterizing the antiproliferative/proapoptotic properties of the CB_1_ agonist ACEA, CB_1_ antagonist/inverse agonist AM281, CB_2_ agonist JWH133, and CB_2_ antagonist/inverse agonist AM630 in the three glioblastoma cell lines U-138 MG, U-87 MG, and LN229. Finally, the role of cannabinoid receptor stimulation was examined in a glioblastoma invasion model of organotypic hippocampal slice cultures (OHSC).

## 2. Results

### 2.1. MicroRNA Expression Is Not Affected by Cannabinoids

**Figure 1:**
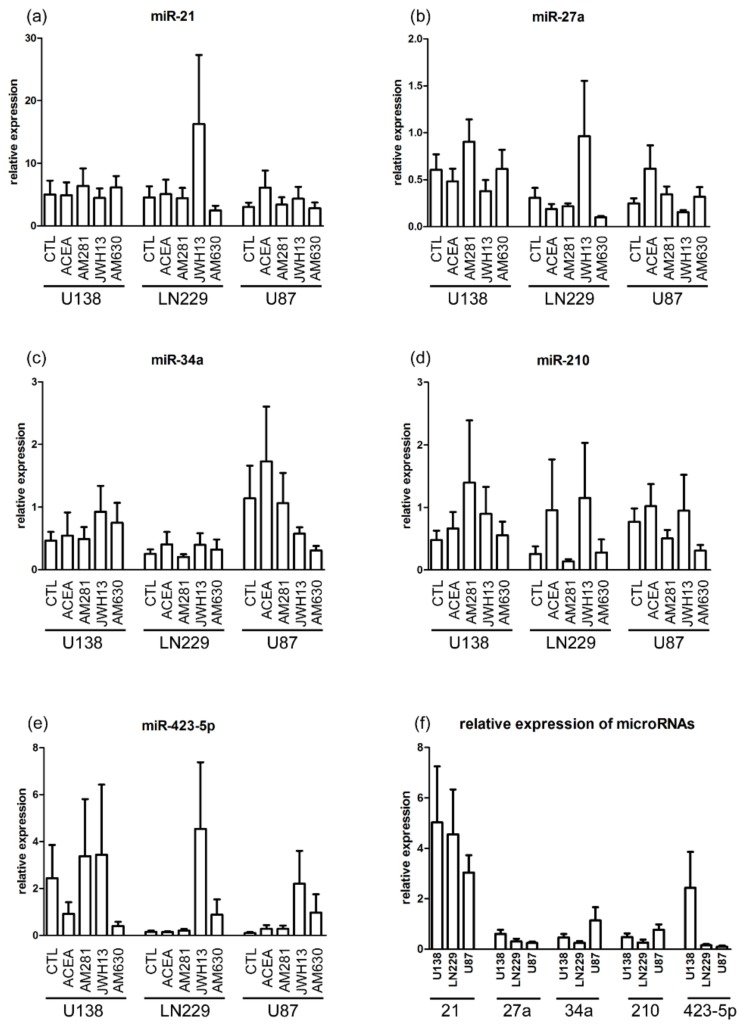
Analysis of the microRNA (miR) profile in glioblastoma cell lines after treatment with agonists (ACEA, 10 µM; JWH133, 10 µM) and antagonists (AM281, 1 µM; AM630, 1 µM) for cannabinoid receptors (CB)_1_ and CB_2_. Each sample was run in duplicates and quantified by 2^−ΔΔC^_T_ (DCT) method. Small RNAs, SNORD44 and let-7a, were chosen as reference genes and serve as a reference according to a NormFinder analysis. (**a**) Expression of miR-21 in U-138 MG (*n* = 6–8), LN229 (*n* = 7–8) and U-87 MG (*n* = 9–10). (**b**) Expression of miR-27a in U-138 MG (*n* = 6–7), LN229 (*n* = 6–8) and U-87 MG (*n* = 9–10). (**c**) Expression of miR-34a in U-138 MG (*n* = 6–7), LN229 (*n* = 6–8) and U-87 MG (*n* = 9–10). (**d**) Expression of miR-210 in U-138 MG (*n* = 6–7), LN229 (*n* = 6–8) and U-87 MG (*n* = 8–10). (**e**) Expression of miR-423-5p in U-138 MG (*n* = 5–7), LN229 (*n* = 5–7) and U-87 MG (*n* = 9–10). (**f**) No significant differences could be observed in the expression of miRs 21, 27a, 34a, 210, and 423-5p between the control groups.

All examined miRs, miR-21, miR-27a, miR-34a, miR-210, and miR-423-5p were found in the cell lines U-138 MG, LN229, and U-87 MG. The expression of miR-21, miR-27a, miR-34a, miR-210, and miR-423-5p in U-138 MG, LN229, and U-87 MG was measured and no significant changes after 24 h treatment with ACEA (10 µM), AM281 (1 µM), JWH133 (10 µM), and AM630 (1 µM) were detected (Figure 1a–f).

### 2.2. Cannabinoids Do Not Influence Proliferation and Cell Death of Glioblastoma Cell Lines

**Figure 2:**
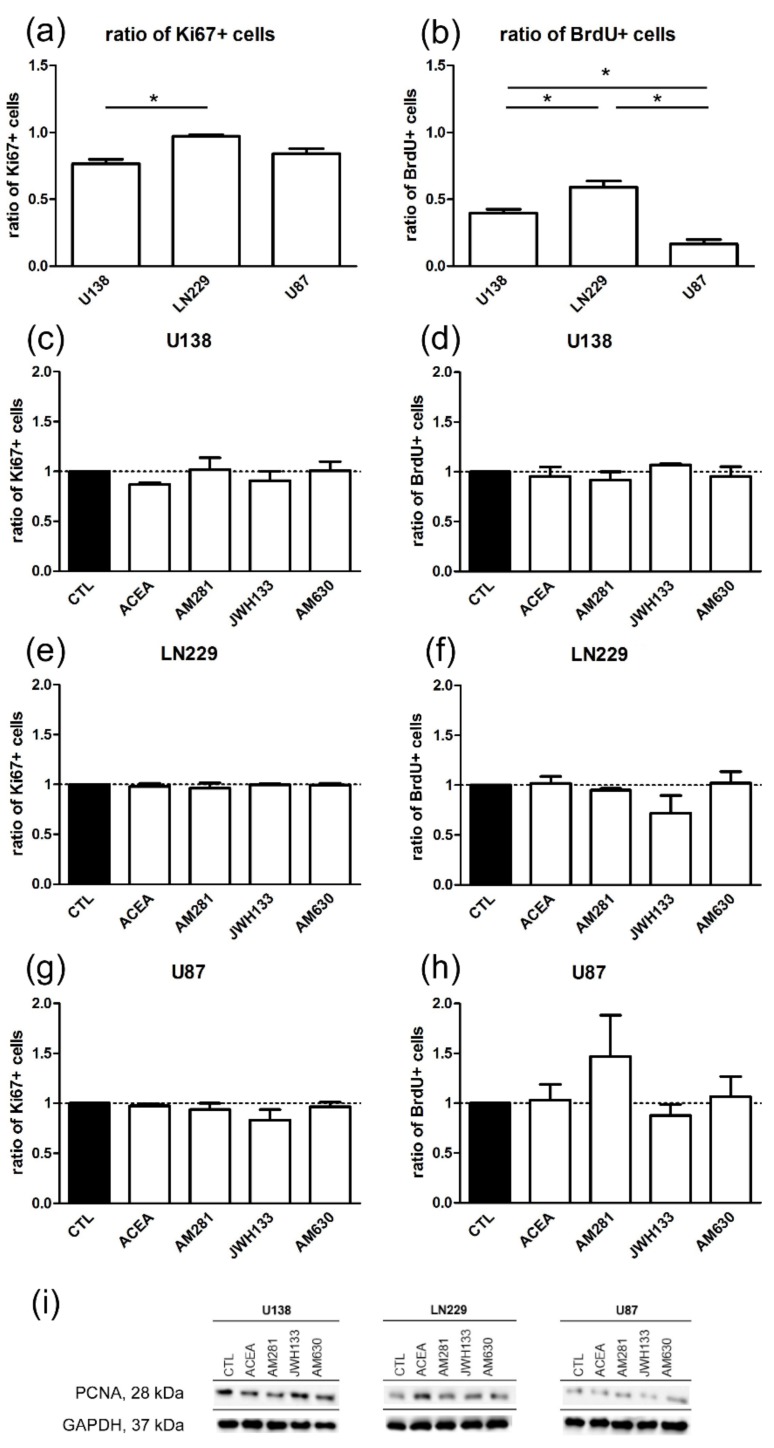
No changes in the proliferation index could be observed in U-138 MG, LN229, and U-87 MG cell lines after treatment with agonists (ACEA, 10 µM; JWH133, 10 µM) and antagonists (AM281, 1 µM; AM630, 1 µM) for CB_1_ and CB_2_ for 24 h. Differences occurred in the basal level of proliferation between the cell lines. Control groups of U-138 MG, LN229, and U-87 MG cell lines were compared in the ratio of positive cells for (**a**) Ki67 (*n*_U138_ = 4, *n*_LN229_ = 3, *n*_U87_ = 4) and (**b**) bromodeoxyuridine (BrdU) (*n*_U138_ = 3, *n*_LN229_ = 4, *n*_U87_ = 3). All cell lines had a significantly different expression of S-phase marker Brdu. The Ki67 index was significantly different between U-138 MG and LN229. LN229 cells have the highest level of proliferating cells followed by U-138 MG. Application of ACEA, AM281, JWH133, and AM630 did not influence the ratio of the BrdU and Ki67 positive cells in (**c**,**d**) U-138 MG (*n*_CTL_ = 3, *n*_ACEA_ = 3, *n*_AM281_ = 3, *n*_JWH133_ = 3, *n*_AM630_ = 3); (**e**,**f**) LN229 (*n*_CTL_ = 3, *n*_ACEA_ = 3, *n*_AM281_ = 3, *n*_JWH133_ = 3, *n*_AM630_ = 3); and (**g**,**h**) U-87 MG cells (*n*_CTL_ = 3, *n*_ACEA_ = 3, *n*_AM281_ = 3, *n*_JWH133_ = 3, *n*_AM630_ = 3). (**i**) Representative Western Blots of G1 and S-phase marker, proliferating nuclear antigen (PCNA) and glyceraldehyde 3-phosphate dehydrogenase (GAPDH) show no significant differences between the groups. All data were normalized to the control group.

To study the changes in proliferation of cell lines, three different markers, namely Ki67, bromodeoxyuridine (BrdU), and proliferating nuclear antigen (PCNA), were examined 24 h after incubation with cannabinoids according to an earlier study demonstrating significant effect on the invasive capacity of these tumor cells [15]. Ki67 is expressed during the whole cell cycle, except for G0, in the nucleus, whereas BrdU, is incorporated during the S-phase only. Proliferating nuclear antigen is expressed during early G1 and S-phase and is essential for replication as a cofactor of DNA polymerases [36]. U-138 MG and LN229 cells differed regarding their portion of Ki67 positive cells (U-138 MG:0.77 ± 0.06; LN229:0.97 ± 0.02; U-87 MG:0.84 ± 0.08), while the ratio of BrdU positive cells was significantly different between all cell lines (U-138 MG:0.40 ± 0.05; LN229:0.59 ± 0.05; U-87 MG:0.17 ± 0.06) (Figure 2a,b). No changes in the expression of Ki67, S-phase marker BrdU or G1, and S-phase marker PCNA was detected after 24 h treatment with ACEA, AM281, JWH133, or AM630 in all cell lines (Figure 2c–i). All results were normalized to the control group of the same cell line. 

**Figure 3:**
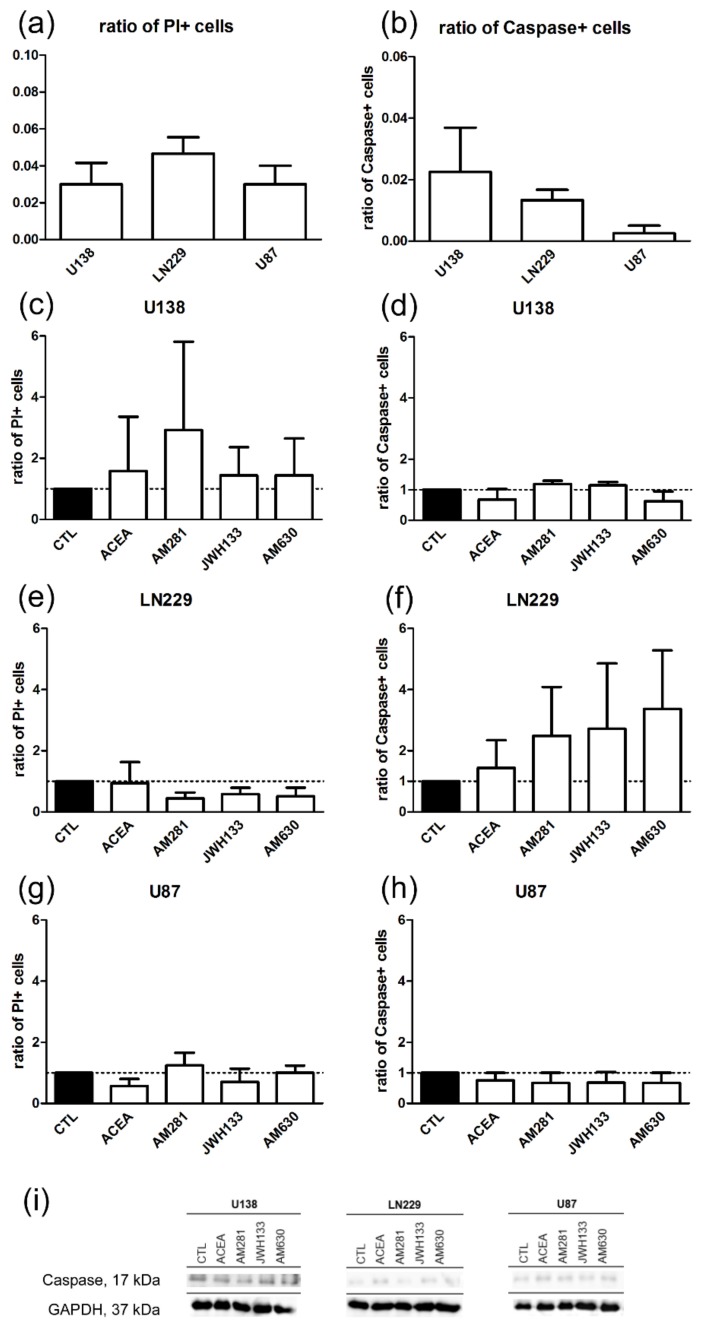
No changes in the ratio of death cells could be observed in U-138 MG, LN229, and U-87 MG cell lines after treatment with agonists (ACEA, 10 µM; JWH133, 10 µM) and antagonists (AM281, 1 µM; AM630, 1 µM) for CB_1_ and CB_2_. Differences between the cell lines in the basal level of cell death markers were not significant. Control groups of U-138 MG, LN229 and U-87 MG cell lines were compared in the ratio of positive cells for (**a**) propidium iodide (PI) (*n*_U138_ = 3, *n*_LN229_ = 3, *n*_U87_ = 3) and (**b**) caspase (*n*_U138_ = 4, *n*_LN229_ = 3, *n*_U87_ = 4). Application of ACEA, AM281, JWH133 and AM630 did not influence the ratio of PI or caspase positive cells in (**c**), (**d**) U-138 MG (*n*_CTL_ = 3, *n*_ACEA_ = 3, *n*_AM281_ = 3, *n*_JWH133_ = 3, *n*_AM630_ = 3); (**e**), (**f**) LN229 (*n*_CTL_ = 3, *n*_ACEA_ = 3, *n*_AM281_ = 3, *n*_JWH133_ = 3, *n*_AM630_ = 3) and (**g**), (h) U-87 MG cells (*n*_CTL_ = 3, *n*_ACEA_ = 3, *n*_AM281_ = 3, *n*_JWH133_ = 3, *n*_AM630_= 3). (**i**) Representative Western Blots of caspase and GAPDH show no significant differences between the groups. All data were normalized to control group.

To additionally assess cell death, cleaved caspase-3 and propidium iodide (PI) were used as cell apoptosis and necrosis markers respectively. Cleaved caspase-3 is one of the key proteases involved in apoptosis [37]. Propidium iodide binds to nucleic acids and if applied prior to fixation of cells with membrane damage it enters the cells and binds to DNA or RNA fragments. Therefore, PI is usually used for visualizing cell death. The basal ratios of caspase positive cells or PI positive cells were very low (<10%). No significant differences were found between the cell lines in the ratio of death PI positive (U-138 MG:0.03 ± 0.02; LN229:0.05 ± 0.02; U-87 MG:0.03 ± 0.02) or apoptotic caspase positive cells between the cell lines (U-138 MG:0.02 ± 0.03; LN229:0.01 ± 0.01; U-87 MG:0.003 ± 0.003) (Figure 3a,b).

After application of CB agonists/antagonists no alterations were observed in the ratio of death PI positive cells in all cell lines. Apoptosis markers in immunohistochemical analysis and in Western Blot were not significantly affected in glioblastoma cells after treatment with ACEA, AM281, JWH133, and AM630 in U-138 MG, LN229, and U-87 MG (Figure 3c–i).

### 2.3. Synthetic Cannabinoids Do Not Activate Akt in Glioblastoma Cells

**Figure 4:**
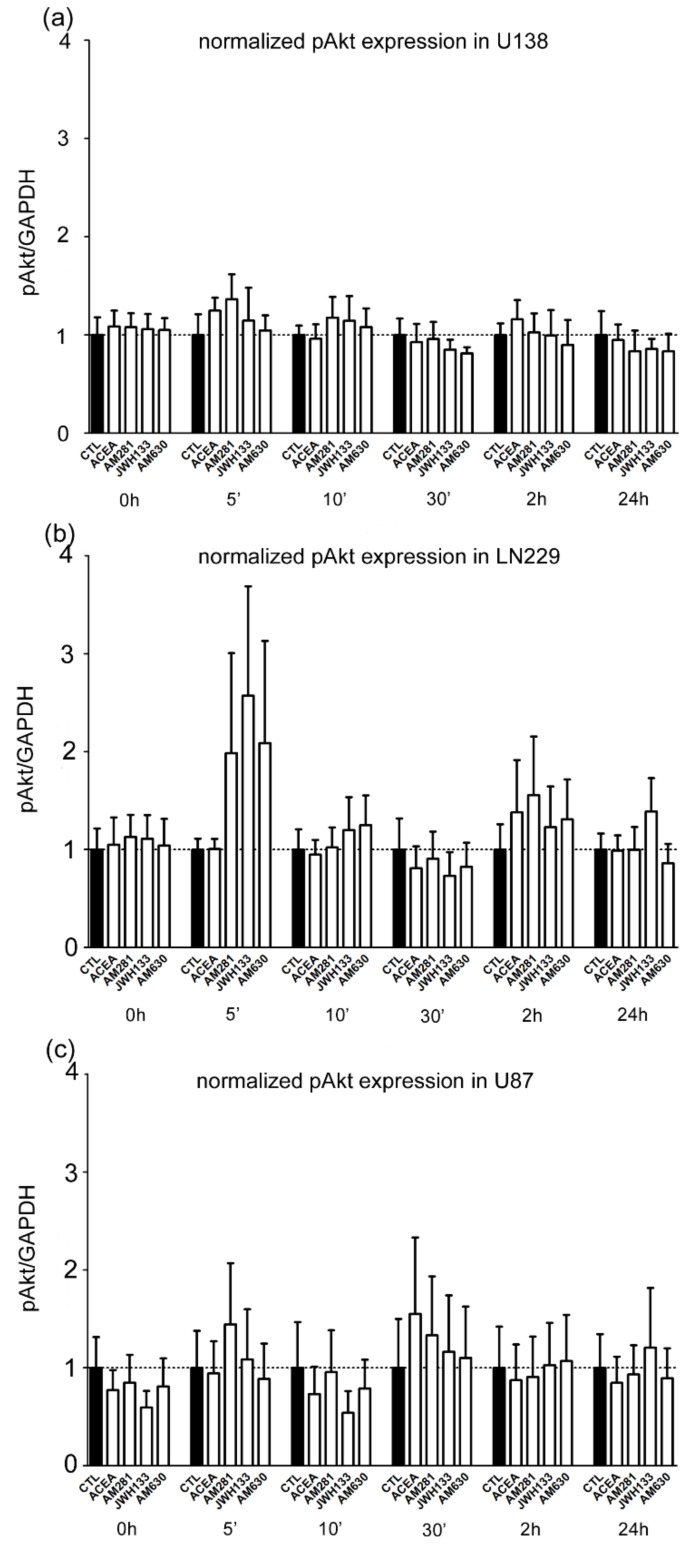
Activation of PI3K-Akt pathway in glioblastoma cell lines (**a**) U-138 MG, (**b**) LN229, and (**c**) U-87 MG. After 0 min, 5 min, 10 min, 30 min, 2 h, and 24 h no significant changes were observed in all examined cell lines (U-138 MG: *n* = 5–7, LN229: *n* = 5–9, U-87 MG: *n* = 4–7) in groups treated with agonists (ACEA, 10 µM; JWH133, 10 µM) and antagonists (AM281, 1 µM; AM630, 1 µM) for CB_1_ and CB_2_.

Analysis of activation of the Akt signaling pathway after cannabinoid treatment with ACEA, AM281, JWH133, or AM630 showed no significant effect at the investigated time points in comparison to the control group (Figure 4a–c). In all three cell lines the level of pAkt normalized to glyceraldehyde 3-phosphate dehydrogenase (GAPDH) remained constant after 5 min, 10 min, 30 min, 2 h, and 24 h. 

### 2.4. Cannabinoids Affect Invasion through Specific Receptors

**Figure 5:**
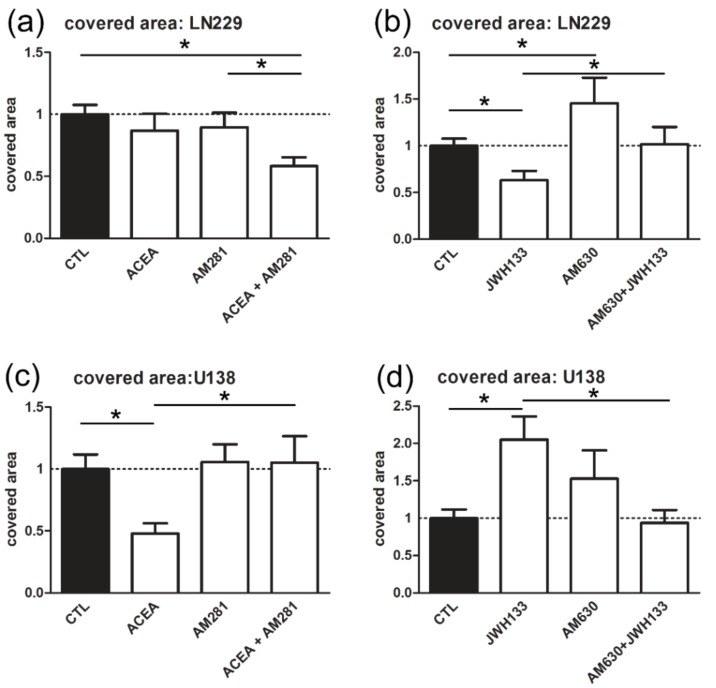
Invasiveness of glioblastoma cells was analyzed in a co-culture model with murine organotypical slice cultures. (**a**,**b**) Treatment with AM281 (1 µM) had no significant effect on the covered area, whereas coincubation of AM281 with ACEA (10 µM) led to strong anti-invasive effect in LN229. Application of AM630 (1 µM) alone led to significant increase in invasiveness of LN229. Treatment with combination of AM630 with JWH133 reversed the JWH133 (10 µM) mediated reduction in invasiveness indicating that previously observed decrease in covered area was mediated by CB_2_ receptor (LN229: *n*_LN229 CTL_ = 60, *n*_LN229 ACEA_ = 34, *n*_LN229 AM281_ = 33, *n*_LN229 ACEA+AM281_ = 35, n_LN229 JWH133_ = 18, *n*_LN229 AM630_ = 19, *n*_LN229 JWH133+AM630_ = 15). (**c**,**d**) Coincubation of ACEA and AM281 reversed the decrease in the covered area mediated by ACEA in U138-MG cells. Incubation with JWH133 led to an increase in the invasiveness, which was abolished by co-application with AM630 (U-138 MG: *n*_U138 CTL_ = 69, *n*_U138 ACEA_ = 23, *n*_U138 AM281_ = 32, *n*_U138 ACEA+AM281_ = 21, *n*_U138 JWH133_ = 32, *n*_U138 AM630_ = 24, *n*_U138 JWH133+AM630_ = 25).

Treatment with CB_1_ antagonist AM281 (AM281: 0.89 ± 0.12) or CB_1_ agonist ACEA (0.86 ± 0.14) had no significant effect on the invasiveness of LN229 when compared to the control (1 ± 0.08), whereas coincubation of AM281 with ACEA (0.58 ± 0.07) induced a strong anti-invasive effect. CB_2_ agonist JWH133 (0.63 + 0.10) reduced the invasiveness of LN229 cells, being antagonized by additional application of AM630 (JWH133 + AM630: 1.02 ± 0.18). Blockade of CB_2_ with AM630 (1.45 ± 0.27) alone increased the invasiveness of LN229 (Figure 5a,b).

In the case of U-138 MG cells (CTL: 1 ± 0.11), ACEA and JWH133 generated very similar effects as previously published [15]. The CB_1_ agonist (ACEA: 0.48 ± 0.24) had anti-invasive effect, while the CB_2_ agonist (JWH133: 2.05 ± 0.31) induced a strong increase in the covered area. The previously reported reduction in the covered area was attenuated by coincubation with AM281 (ACEA + AM281: 1.05 ± 0.21), whereas AM281 alone (AM281: 1.06 ± 0.14) had no effect on tumor invasion. The effect of JWH133 (2.05 ± 0.31) was reversible if coincubated with AM630 (JWH133 + AM630: 0.94 ± 0.17). AM630 alone (AM630: 1.53 ± 0.38) had no significant effect on the invasiveness (Figure 5c,d).

## 3. Discussion

Glioblastomas are the most aggressive tumors of the central nervous system. In solid glioblastoma heterogeneous areas were found, the tumor is characterized on the one hand by its highly proliferative nature and on the other hand by necrotic and apoptotic regions [38]. Additionally, the wide range of different tumors—which are classified as glioblastoma grade IV by the World Health Organization (WHO)—makes it difficult to provide reliable results valid for all cases [39]. Due to its high resistance against standard therapies, an improvement of current treatments is of high importance. One potential additional therapeutic approach are cannabinoids, which were shown to affect tumor cells in different models with and without toxic effects to healthy tissue [10,40,41]. In previous studies an anti-invasive effect of cannabinoids in glioblastoma cell lines was observed as cannabinoids changed motility, inhibited proliferation, activated apoptosis, and blocked cell cycle progression [10,15,42,43,44]. Since glioblastoma is known to be very heterogeneous [45,46], differences between cell lines were observed and mechanisms behind cannabinoid mediated actions are not fully understood. Even less clear is the impact of specifically targeting CB_1_ and CB_2_ receptors. Therefore, we studied the effect of synthetic cannabinoids with high selectivity to CB_1_ and CB_2_ receptors on miRs expression, proliferation, apoptosis, pAkt activation, and invasiveness in three significantly different glioblastoma cell lines [47,48,49,50].

### 3.1. Cannabinoid Stimulation Does Not Influence microRNAs Expression in Glioblastomas

Amongst others we examined possible cross-talk between miRs and cannabinoids in glioblastoma cell lines. miRs contribute to the maintenance and phenotype of different tumor cells [51]. As reported recently, a single miR can target different messenger RNAs (mRNAs), and one mRNA can be influenced by various miRs [51].

All of the examined miRs have previously been shown to target different mRNAs important for survival, proliferation, apoptosis, invasion, cytoskeletal rearrangement and/or migration, and angiogenesis of tumor cells and glioblastoma [24,52,53]. Since cannabinoids influence the invasiveness and other processes in glioblastoma cells [15,54], the effects on common miRs in glioblastomas were examined. The following miRs were upregulated or downregulated in glioblastomas; miR-21, miR-210, miR-34a, miR-27a, and miR-423-5p. All these miRs have different targets and are important for control of cell proliferation and tumor growth, regulation of apoptosis and are potential noninvasive biomarkers for diagnosis and prognosis of gliomas [24,55,56,57,58]. In different tumor types, but not in glioblastomas, it was shown that CB and miRs can influence each other. miR-27a was partly induced by the CB_1_/CB_2_ agonist WIN 55,212-2 in colon carcinoma cell lines [31], and let-7d was shown to be a target of CB_1_ controlling cannabinoid signaling in SH-SY5Y neuroblastoma cells [34]. Furthermore, cross-talk between miRs and signaling cascades was reported. miR-34a suppressed cell proliferation and tumor growth of glioma stem cells by targeting Akt and Wnt signaling [24,56]. In contrast to these findings, no significant effect was found in the expression of miR-21, -27a, -34a, -210, and -423-5p after cannabinoid treatment in this study. However, basic levels between miRs in cell lines were different. One potential reason for the absence of any observable interaction between cannabinoids and miRs might be that another of 200 miRs that are differently expressed in glioblastoma is targeted [24]. It should also be considered that cannabinoids are supposed to trigger signaling cascades on a very short time scale, but the resulting effects last significantly longer [59,60,61,62]. Therefore, the changes of miRs might occur on a comparable time scale that we could not resolve here, since some miRs are stable for more than 24 h and others between 4 and 14h only [63]. Nevertheless, for the chosen miRs it is unlikely that cannabinoid receptor activation or blockade modulates their expression.

### 3.2. Modulation of Cell Proliferation and Apoptosis by Cannabinoids

Cannabinoids were shown to exert antiproliferative and proapoptotic effects before [64]. Most studies so far used phytocannabinoids like THC, CBD, or a combination of the two, but both are not specific and target further receptors beyond the classical CB_1_ and CB_2_. Furthermore, studies using the same cell lines observed different effects after cannabinoid treatment in breast and lung carcinomas [65,66,67]. Δ-9-tetrahydrocannabinol induced apoptosis in C6.9 glioma cells after 5 days in serum free medium independent of CB_1_ [68], while JWH133 caused apoptosis via CB_2_ [69]. In our study neither CB_1_ nor CB_2_ ligands induced glioblastoma cell death, but they significantly affected the invasiveness in the OHSC model. Δ-9-tetrahydrocannabinol was shown to block glioblastoma progression by influencing various S-phases of the cell cycle in U-251 MG and U-87 MG cells [44]. In U-251 MG cells THC and CBD led to cell cycle arrest in G0-G1 phase, but not G2-M, whereas co-application of THC and CBD resulted in an additive increase in the population of cells in G0-G1 and G2-M phase and a reduction in S-phase [14]. In contrast, we could not observe any cell cycle arrest/reduction in G0 (Ki67 labeling), S-phase (BrdU staining) or G1/S (PCNA labeling) after incubation with the specific and selective cannabinoid agonists and antagonists ACEA, JWH133, AM281, and AM630. Opposite to the phytocannabinoids THC and CBD, ACEA and JWH133 are full agonists at CB receptors. Δ-9-tetrahydrocannabinol is only a partial agonist of CB receptors with an affinity and efficacy for CB_1_ and CB_2_ (K_i_ = 5.05; 3.13 nM, respectively) as well as for GPR55 receptor (EC_50_ GPR55 = 8 nM). CBD is reported to be a weak CB_1_ antagonist, a CB_2_ inverse agonist (IC_50_ = 0.445; 3.35 μM), and a weak agonist at VR1 vanilloid receptors [9,70,71]. It seems therefore conceivable that the previously reported cell cycle effects of THC and CBD are independent of CB_1_ and CB_2_.

An additional difference of measured proliferative effects may arise from the assay, because many studies used the MTT assay, being an indirect way of measuring proliferation [72]. In contrast, staining and microscopic analysis is considerably more reliable. Another possible explanation is that the exposition time was too short. The incubation time differed between previous and the current study, as we used an incubation time of 1 day, while in previous studies cells were often incubated up to 5 days [14,68]. Here, cells were incubated for 24 h since cannabinoids after 24 h induced significant effects on the invasion [15]. A longer incubation time might also lead to receptor desensitization, causing measured effects to be more likely not be mediated via classical cannabinoid receptors [73]. Many studies investigating the effect of cannabinoids on proliferation, apoptosis, etc. use serum starved cells for their experiments, potentially being the cause for the nonexistent regulation of proliferation and apoptosis observed in this study; however, this is not comparable with the in vivo situation, when glioblastomas are supplied by leaky blood vessels. Different studies thereby observed that THC, anandamide, and CBD effects on apoptosis were inhibited by the addition of serum [74,75]. Nevertheless, in this and a previous study of our lab we found cannabinoid agonists to induce effects on the invasive capabilities of the used glioblastoma cell lines in serum containing medium [15], suggesting the effectiveness of the used culture conditions and concentrations to influence biological behavior. Given the heterogeneity of glioblastoma in vivo it seems necessary to use different culture conditions for drug screening to evaluate effects as it is not intrinsically clear which medium composition mimics intra-tumoral conditions best and thus different culture conditions may approximate different tumor niches [76]. Consequently, different culture conditions may shed light on the spectrum of applicability of cannabinoids.

### 3.3. pAkt Is Not Affected in Glioblastoma Cell Lines after Cannabinoid Treatment

Since cannabinoid receptors are coupled to the PI3K-Akt survival pathway, they can consequently phosphorylate and inhibit nuclear translocation of transcription factors, thereby preventing the expression of proapoptotic proteins [77]. In the present study, we examined the activation of Akt signaling by measuring the amount of phosphorylated Akt. Incubation with synthetic cannabinoids did not result in a significant alteration in the amount of active Akt, when compared to the respective control measurement. Therefore, the previous reported action of ACEA and JWH133 on Akt signaling in differentiating oligodendrocytes or THC and methanandamide in prostate carcinoma PC-3 cells [78,79] could not be reproduced in glioblastoma cell lines. 

Ligand-induced desensitization or internalization of the CB is a plausible explanation for missing effects on pAkt, because both phenomena can be observed already a few hours after application of CB ligands [80,81]. It is known that the level of CB receptor expression determines if the Akt pathway is activated upon cannabinoid stimulation and high expressed CBs are coupled to Akt [16]. Furthermore, mutations, overexpression or downregulation of PTEN (phosphatase and tensin homolog on chromosome 10) or EGFR (epidermal growth factor (EGF) receptor), both common in glioblastomas, are possible modulators of Akt activation or negative regulation [82,83,84]. However, the role of PI3K and Akt in glioblastomas is established, as one of the most important signaling pathways for tumor transformation [85,86]. In this study further mechanisms seem responsible for cannabinoid mediated effects, like Raf-1/ERK/MAP kinase pathway, Wnt/β catenin signaling, JNK or NFκβ pathway [10], or reactive oxygen species response [87].

Consequently, synthetic cannabinoid ligands can activate other signaling mechanisms leading to changes in invasiveness or the GPR receptors can be coupled to other signaling cascades or undergo homo- or heterodimerization, internalization, clustering, or desensitization [88].

### 3.4. Modulation of Invasion by Cannabinoids Is Drug- and Glioblastoma Cell-Line-Specific

The process of invasion is complex and includes modulation of cell–cell and cell–matrix adhesion, remodeling of the extracellular matrix, and cell migration. The invasion process was often evaluated in matrigels (reconstituted basement membrane) with single cells but this approach is difficult to compare to physiological conditions. In the model used in this study, the invasion process in healthy brain tissue was examined [89]. This is of special importance, because the extracellular matrix in the brain differs largely from other tissues and mainly consists of heparin sulphate proteoglycan, hyaluronic acid, chondroitin sulphate proteoglycan, tenascin R and type IV collagen, and fibronectin in the perivascular area [90]. Also, at cellular level brain tumors and their surrounding are composed of microglia, macrophages, astrocytes, oligodendrocytes, neurons, glial and neuronal progenitors, pericytes, and endothelial cells, creating a complex and strongly interacting system. Thus, the tumor micromilieu plays a crucial role for tumor growth, invasiveness and cell death [38]. Due to this milieu, OHSC can be regarded as more physiological than matrigels. Usually, matrigel plates, which are covered with basement membrane, do not reflect the physiological environment of the brain, since glioblastoma cells do not spread or form metastasis in other tissues outside the central nervous system in vivo [89,91]. In spite of the mentioned advantages the OHSC tumor cell culture model represents a combination of murine and human tissues which harbors limitations as well. Furthermore, due to the differences the direct comparison between matrigel and OHSC is limited.

Both, pro- and anti-invasive effects of the used synthetic cannabinoids in OHSC on the one hand seem to be receptor dependent but on the other hand cell type specific regarding the effects. However, the mechanism remains unclear. In a previous study a single concentration of CB_2_ agonists reduced the invasiveness of LN229 cells and increased the invasiveness of U-138 MG cells [15] and both effects seemed to be mediated by the CB_2_ receptor. In contrast, another glioblastoma cell line (U-87 MG) did not respond to CB_2_ stimulation in a previous study of our lab (see Hohmann et al. 2017 [15]). Coincidentally, CB_2_ receptor antagonism in LN229 increased the invasiveness, also receptor dependent, probably related to the fact that AM630 might be a protean ligand depending on receptor constitutive activity and system [92]. Anti-invasive effects due to CB_2_ activation were observed before. In mouse bearing C6.9 gliomas JWH133 reduced tumor volume [93] and induced regression of C6 cell gliomas in vivo by selective CB_2_ activation [69]. In osteosarcoma JWH133 led to a less invasive phenotype [94]. In U-87 MG the effect of the phytocannabinoid CBD on tumor cell growth was reversed after antagonization of CB_2_ [95]; however, JWH133 alone did not alter the invasiveness [15]. However, other effects (e.g., migration) of CBD on glioblastoma cells seemed to be CB receptor independent [42]. Also, SR144528, a CB_2_ antagonist, and AM251, a CB_1_ antagonist reduced CBD mediated apoptosis in U-118 MG and U-87 MG, whereas AM251 and SR144528 alone had no effect on apoptosis [41]. The effect of CB_2_ agonists seems to be tumor-specific and needs further examination. After application of the CB_1_ agonist ACEA no effect was observed in LN229 or U-87 MG, but in U-138 MG the invasion was reduced in a CB_1_ receptor dependent manner [15]. Coincubation of AM281 with ACEA decreased the invasiveness in LN229.

Considering cannabinoids, it is known that they activate a multitude of different targets, including matrix metalloproteinases, ROS or pathways like mTOR, p38 MAPK, and STAT3 [10]. In contrast, there is—to the authors knowledge—no study examining the effects of specific cannabinoid receptor activation on cells bearing the respective receptor but showing different biological behavior after stimulation, as observed here for example for the effect of JWH133 on the invasiveness of LN229 and U-138 MG cells. Thus, the evaluation of downstream events after cannabinoid treatment is of high importance to shed light on the observed heterogeneous effects of CB stimulation on tumor invasion. Possible starting points are mutations occurring in the used cell lines, like mutation of p53 or PTEN, because the used cell lines differ in both their PTEN and p53 status [82]. Especially the PTEN mutation might be of interest as activated PTEN inhibits Phosphatidylinositol (3,4,5)-trisphosphate (PtdIns(3,4,5)P3) and focal adhesion kinase (FAK) [96,97], both being targets of cannabinoids as well [98,99,100,101]. FAK is directly targeted by cannabinoids while PtdIns(3,4,5)P3 is modulated by PI3K, being a target of cannabinoids as well [98,100,101,102].

Consequently, the following questions are raised. How are the tumor-specific effects mediated? What mechanisms alter the responsiveness of glioblastoma to cannabinoid treatment? It is likely that this heterogeneity influences the impact of cannabinoids as well.

## 4. Materials and Methods

### 4.1. Cell Culture

U-87 MG and LN229 cells were purchased from the American Type Culture Collection (U-87 MG: ATCC HTB-14; LN229: ATCC CRL-261, Manassas, VA, USA) and U-138 MG cells were obtained from Cell Lines Service (Cell Lines Service, 300363, Eppelheim, Germany).

Cell lines were authenticated using Multiplex Cell Authentication by Multiplexion (Heidelberg, Germany) as described recently [103]. The single nucleotide polymorphism (SNP) profiles matched known profiles or were unique.

All cell lines were cultured as described elsewhere [15]. Twenty-four hours prior to the start of experiments the culture medium was changed, and cannabinoids were added to the respective groups. We used the CB_1_ agonist ACEA (10 µM, solved in ethanol; Tocris, 12A/141371, Minneapolis, MN, USA), the CB_1_ antagonist/inverse agonist AM281 (1 µM, solved in dimethyl sulfoxide (DMSO), Tocris, 1115), the CB_2_ agonist JWH133 (10 µM, solved in DMSO; Tocris, 5B/97327), and the CB_2_ antagonist/inverse agonist AM630 (1 µM, solved in DMSO, Tocris, 1120). The same concentrations of cannabinoids were used throughout the whole study.

For Western Blot and miR experiments, 400,000 cells were seeded in 6-well plates and incubated overnight in medium. On the next day cells were treated for up to 24 h with cannabinoids. The cells were detached using Trypsin/EDTA (Merck Millipore, Billerica, MA, USA) and stored at −80°C. 

For immunohistochemical analysis, 50,000 cells were placed on glass cover slips covered with poly-l-lysin (Sigma Aldrich, St. Louis, MO, USA). Cannabinoids were applied one day later for 24 h. Bromodeoxyuridine (BrdU) (0.01 mM, B5002, Sigma Aldrich) and propidium iodide (PI, 5 µg/mL, Merck Millipore) were added to the culture medium 6 h or 2 h before the fixation, respectively. Cells were fixed with 4% paraformaldehyde for 10 min and stored in phosphate buffered saline (PBS) at 4°C for further analysis.

### 4.2. Organotypic Hippocampal Slice Cultures

All experiments involving animal material were performed in accordance with the directive 2010/63/EU of the European Parliament and the Council of the European Union (22.09.2010) and approved by local authorities of the State of Saxony-Anhalt (I11M18) protecting animals and regulating tissue collection used for scientific purposes.

Organotypic hippocampal slice cultures were prepared from 5-day-old C57Bl6/J mice as reported earlier [15,89,104] and kept at 35°C in a fully humidified atmosphere with 5% (v/v) CO_2_. Culture medium was changed every other day. After 14 days in vitro the experiments were started. Tumor cells were labeled using the fluorophores carboxyfluorescin diacetate (CFDA; Gibco, Thermo Fisher, Waltham, MA, USA 12883) and slice cultures using PI [15,89,104,105].

### 4.3. Confocal Laser Scanning Microscopy

The analysis of fixed OHSC was performed using a confocal laser scanning microscope (Zeiss LSM710, Carl Zeiss, Jena, Germany) with an excitation wavelength of 488 nm for CFDA and 543 nm for PI. Emission was detected using a band-pass filter sensitive for Δλ = 510–550 nm (CFDA) and Δλ = 610–720 nm (PI). The tumor invasion pattern was visualized with a 10x objective, as a z-stack with a step width of 2 µm. The obtained image stack was analyzed using the maximal intensity projection and the application of a threshold algorithm to calculate the area of the OHSC covered by tumor cells. The invasiveness was normalized to the invasiveness of the respective control group.

### 4.4. Immunohistochemistry

Glioblastoma cells were stained as published before [104]. Ki67 was used for the assessment of proliferation, and cleaved caspase 3 for apoptosis detection (Table 1). The labeling of incorporated BrdU was visualized with an anti-BrdU antibody (1:100, DAKO, Aligent, Santa Clara, CA, USA) (Table 1), which was applied for 1 h. For all antibodies and all immunohistochemical stainings, the subsequent steps were identical. After washing with PBS, a biotinylated goat anti-rabbit antibody (1:100, Sigma Aldrich) was applied for one hour, the cells were washed three times with PBS and incubated with Streptavidin for one hour (1:100, Sigma Aldrich). After washing with PBS and Tris buffer, the slides were stained with DAB (Sigma Aldrich) and Hematoxylin (Merck Millipore) and covered with Entallan (Merck Millipore). Propidium iodide-labeled cells were additionally stained with Sytox Green (1:10 000, S7020, Thermo Fisher) for 5 min before covering with DAKO mounting medium (DAKO).

For assessing cell death and proliferation at least 100 cells were counted and 5 areas for each cover slip were recorded with an Axioplan (Zeiss, Oberkochen, Germany) and analyzed using ImageJ v1.46r (National Institutes of Health, Laboratory for Optical and Computational Instrumentation, University of Wisconsin, Madison, WI, USA).

### 4.5. Western Blot

The analysis was performed as described before [15,104]. Ten micrograms of the sample was loaded on an electrophorese gel. Anti-phospho-Akt (Cell Signaling, 1:2000) (Table 1) and anti-GAPDH (Cell Signaling, 1:1000) antibodies were used for the analysis of signaling cascade and as loading control. For the assessment of proliferation and apoptosis, horseradish peroxidase (HRP)-conjugated PCNA (Santa Cruz, Santa Cruz, CA; USA) and cleaved caspase 3 (Cell Signaling) were used, respectively. The imaging and evaluation of blots was performed with a Fusion FX7 (PeqLab, VWR, Kelsterbach, Germany).

### 4.6. RNA Extraction and DNase Treatment

The isolation of RNA and DNase treatment were performed as described before [15]. RNA was isolated using the Trizol (PeqLab) method and with the DNA-freeTM Kit (Promega, Madison, WI, USA).

### 4.7. Analysis of microRNAs expression

MicroRNA quantification was performed as described previously [35]. Briefly, for each sample, 40 ng of RNA was transcribed to complementary DNA (cDNA) applying miRCURY Universal cDNA Synthesis kit II (Exiqon, Verdbaek, Denmark) according to the manufacturer’s protocol. cDNA was diluted 20-fold in RNAse-free water and analyzed in a quantitative real-time PCR cycler (MyIQ cycler, Biorad, Hercules, CA, USA), using miRCURY ExiLENT SYBR Green master mix (Exiqon, Verbaek, Denmark). Each sample was run in duplicates and quantified by 2^−ΔΔC^_T_ (DCT) method [106]. Small RNAs SNORD44 and let-7a were chosen as reference genes according to a NormFinder analysis.

### 4.8. Statistical Analysis

Statistics was performed using the one-way ANOVA with Dunnet test or *t*-test and significance was chosen for *p* < 0.05. Grubbs’ test was used for detection of outliners. All *p*-values refer to the respective controls of the same parameter of the same cell line or to the treatment with the agonist for the respective receptor.

## 5. Conclusions

The effects of synthetic cannabinoid agonists and antagonists on different glioblastoma cell lines were compared. Cannabinoid mediated anti- and pro-invasive effects have been observed in the current and in previous studies [7,107]. A cannabinoid induced expression of miRs, proliferation, apoptosis and pAkt activation could not be found. We also evaluated effects of the selective CB_1_ ligands ACEA and AM281 and CB_2_ ligands JWH133 and AM630 on invasiveness, which were cell- and receptor-dependent. It is well known that for standard therapy with temozolomide there is a population of responders and nonresponders [1] and a similar mechanism seems to occur with cannabinoids. As observed here and in a previous study of our lab, there are three populations of glioblastoma cells: nonresponders, positive responders (reduced invasiveness), and negative responders (increased invasiveness) [15]. This heterogeneous response was observed even though the cell lines possessed the respective receptor. Consequently, receptor presence is not a sufficient explanation for the observed effects and downstream targets and receptor dimerization of cannabinoids need to be evaluated. Other mechanisms like alterations of cell–cell and cell–matrix adhesion, remodeling of the extracellular matrix, and cell migration could be responsible for these effects and need further examination. Nevertheless, due to the short survival time of patients and resistance to standard therapy, cannabinoids should be considered as a promising drug group for patients with glioblastomas, if cannabinoid sensitivity is given.

## Figures and Tables

**Table 1:** Antibodies and fluorophores.

Antibodies and Fluorophores	Species	Company	Concentration	Article Number
**Microscopy**				
anti-Ki67	rabbit	DSC innovative Diagnostik-System	1:200	KI681C002
anti-BrdU	mouse	Dako	1:100	M0744
PI	-	Sigma Aldrich	5 µg/mL	P4170
anti-rabbit IgG, biotin conjugated	goat	Sigma Aldrich	1:100	B7389
anti-rabbit IgG, horseradish peroxidase (HRP)-conjugated	goat	Vector	1:20 000	A0545
anti-mouse IgG, biotin conjugated	goat	Sigma Aldrich	1:100	B7264
**Western Blot**				
anti-PCNA	mouse	Santa Cruz	1:1000	sc-56
anti-cleaved caspase 3 (Asp175/5A1E)	rabbit	Cell Signaling	1:2000	9664
anti-GAPDH (14C10)	rabbit	Cell Signaling	1:1000	2118
p-Akt (Ser473)	rabbit	Cell Signaling	1:2000	9271
